# Atomic Layer Deposition of Ultrathin La_2_O_3_/Al_2_O_3_ Nanolaminates on MoS_2_ with Ultraviolet Ozone Treatment

**DOI:** 10.3390/ma15051794

**Published:** 2022-02-27

**Authors:** Jibin Fan, Yimeng Shi, Hongxia Liu, Shulong Wang, Lijun Luan, Li Duan, Yan Zhang, Xing Wei

**Affiliations:** 1 School of Materials Science and Engineering, Chang’an University, Xi’an 710061, China; 2019131040@chd.edu.cn (Y.S.); lijunl@chd.edu.cn (L.L.); liduan@chd.edu.cn (L.D.); yan.zhang@chd.edu.cn (Y.Z.); weixing_b319@163.com (X.W.); 2 Key Laboratory of Wide Band-Gap Semiconductor Materials and Devices, School of Microelectronics, Xidian University, Xi’an 710071, China; hxliu@mail.xidian.edu.cn (H.L.); slwang@xidian.edu.cn (S.W.)

**Keywords:** atomic layer deposition, MoS_2_, La_2_O_3_/Al_2_O_3_, ultraviolet ozone

## Abstract

Due to the chemically inert surface of MoS_2_, uniform deposition of ultrathin high-κ dielectric using atomic layer deposition (ALD) is difficult. However, this is crucial for the fabrication of field-effect transistors (FETs). In this work, the atomic layer deposition growth of sub-5 nm La_2_O_3_/Al_2_O_3_ nanolaminates on MoS_2_ using different oxidants (H_2_O and O_3_) was investigated. To improve the deposition, the effects of ultraviolet ozone treatment on MoS_2_ surface are also evaluated. It is found that the physical properties and electrical characteristics of La_2_O_3_/Al_2_O_3_ nanolaminates change greatly for different oxidants and treatment processes. These changes are found to be associated with the residual of metal carbide caused by the insufficient interface reactions. Ultraviolet ozone pretreatment can substantially improve the initial growth of sub-5 nm H_2_O-based or O_3_-based La_2_O_3_/Al_2_O_3_ nanolaminates, resulting in a reduction of residual metal carbide. All results indicate that O_3_-based La_2_O_3_/Al_2_O_3_ nanolaminates on MoS_2_ with ultraviolet ozone treatment yielded good electrical performance with low leakage current and no leakage dot, revealing a straightforward approach for realizing sub-5 nm uniform La_2_O_3_/Al_2_O_3_ nanolaminates on MoS_2_.

## 1. Introduction

Silicon complementary metal-oxide-semiconductor (CMOS) devices continuing shrink in size, and keeping the generation of heat low has becoming extremely challenging [1,2]. One promising alternative approach is to use transition metal dichalcogenides (TMDs) due to their extraordinary electronic and mechanical properties [3,4]. Particularly, molybdenum disulfide (MoS_2_) with a natural bandgap (1.2~1.8 eV) has attracted plenty of researches for its promising application in scaled low-power field effect transistors (FETs) and flexible devices [5,6]. A crucial step in the manufacturing of FETs is the growth of ultrathin and uniform high-k gate dielectric on MoS_2_. The mobility of MoS_2_ can be further improved after high-k gate films deposition through the suppression of Coulomb scattering by the dielectric mismatch effect between the MoS_2_ and high-k dielectric [7,8]. The most controlled approach for obtaining nanoscale, high-quality growth of dielectrics is atomic layer deposition (ALD). However, atomic layer deposition of ultrathin and uniform high-k gate films on MoS_2_ still represents one of the key challenges to be addressed due to the lack of dangling bonds or nucleation sites on the MoS_2_ surface. The physical adsorption of precursors on the surface is considered to be a key element that enables the initial ALD reaction to take place [9]. Nevertheless, the weakly physical adsorption of precursors can be easily desorbed from the surface by the subsequent purge gas [10]. The dielectric films easy to form pinhole-like defects when it is directly deposited on MoS_2_ due to random nucleation at defects, edges, and impurities, especially the thickness of the dielectric less than a few nanometers [11]. However, to meet the demand of ultra-scaled FETs, the thickness of gate dielectric layer needs to be extremely thin (<5 nm) for sufficient electrostatic coupling of the gate to the semiconducting channel [12].

To cope with these challenges, the pretreatment of a MoS_2_ surface with oxygen plasma [13], introduction of an additional seeding layer [14], ultraviolet ozone (UV-O_3_) [15], water plasma treatment [16] have been demonstrated. Growth of sub-5 nm uniform Al_2_O_3_ film on MoS_2_ has been achieved [12]. La_2_O_3_ has a high dielectric constant (~26), large band gap (~5.8 eV), and the drawback of moisture absorption can be greatly improved by mixed with a less hygroscopic oxide Al_2_O_3_ [17,18]. It has been studied as the candidate gate dielectric in the sub-22 nm technical process node. The La_2_O_3_/Al_2_O_3_ nanolaminate processed film can provide a higher dielectric constant and better leakage current control at the same physical thickness compared to the Al_2_O_3_ film. However, to date, the growth of La-based binary or ternary compounds on MoS_2_ has not been investigated. Therefore, in this paper, the ALD deposition of sub-5 nm La_2_O_3_/Al_2_O_3_ nanolaminates on MoS_2_ is carried out and the properties of La_2_O_3_/Al_2_O_3_ nanolaminates on MoS_2_ are investigated.

## 2. Materials and Methods

**Figure 1:**
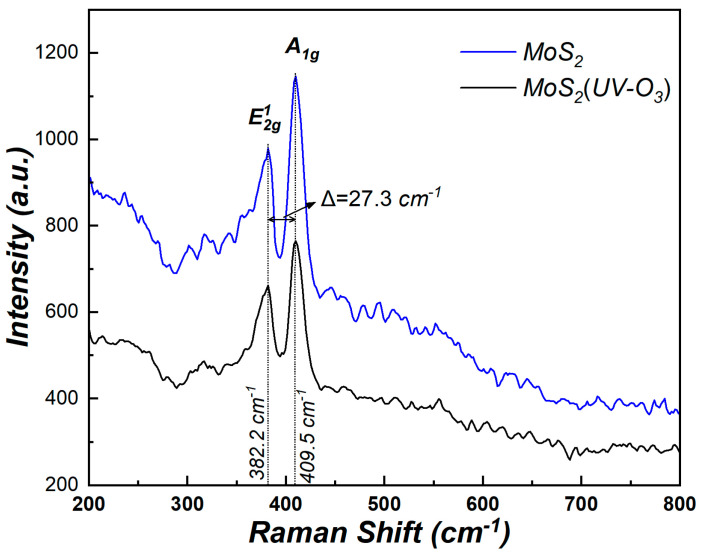
Raman spectra of MoS_2_ before and after UV-O_3_ treatment.

In the experiment, n-type silicon (100) wafers with a resistivity of 2–4 Ω·cm were cleaned by RCA method and a 60 s dip in diluted HF solution was used to remove the native oxide, followed by 5 min of washing with deionized water. Then, the silicon wafers were immediately transferred to an ultra-high vacuum RF magnetron sputtering system chamber and a MoS_2_ target was cleaned in 10 min by pre-sputtering under the deposition conditions. Afterward, few layers MoS_2_ film was directly deposited by RF magnetron sputtering system with the RF power of 50 W at 400 °C. For sulfur compensation and defects reduction, all wafers were annealed in the hydrogen sulfide at 700 °C for 60 min. After that, some wafers were treated by UV ozone ProCleaner plus system under the power of 11.04 mW·m^−2^ for 5 min at the room temperature to improve the surface of MoS_2_. The Raman spectra of MoS_2_ before and after UV-O_3_ treatment is shown in Figure 1. The two characteristics Raman modes (A_1g_ and E^1^_2g_) of the MoS_2_ can be observed and their positions changed negligibly before and after UV-O_3_ treatment. It indicates that the treatment causes minimal structural damage in MoS_2_. Moreover, the difference between these two peaks is 27.3 cm^−1^, which indicated that the thickness of MoS_2_ is between five and seven layers [19].

Then, the wafers with or without UV-O_3_ treatment were transferred to the ALD chamber to deposited La_2_O_3_/Al_2_O_3_ nanolaminates at 260 °C. Tris (isopropylcyclopentadienyl) lanthanum (La(^i^PrCp)_3_) and trimethyl-aluminum (TMA) was used as the lanthanum and aluminum precursor, respectively. H_2_O and O_3_ was used as the oxidant, respectively. O_3_ was generated by the ozone generator using ultra-pure O_2_ (99.999%).10 deposition sequence cycles of TMA/H_2_O/La(^i^PrCp)_3_/H_2_O and TMA/O_3_/La(^i^PrCp)_3_/O_3_ were used to obtain H_2_O-based La_2_O_3_/Al_2_O_3_ nanolaminates and O_3_-based La_2_O_3_/Al_2_O_3_ nanolaminates, respectively. Before the ALD deposition sequence, a 4 s pulse time of TMA was carried out firstly to form the physical adsorption on the surface. ~3 nm H_2_O-based La_2_O_3_/Al_2_O_3_ nanolaminates and O_3_-based La_2_O_3_/Al_2_O_3_ nanolaminates were measured by Woollam M2000D spectroscopic ellipsometry. After O_3_-based and H_2_O-based La_2_O_3_/Al_2_O_3_ nanolaminates deposition process, Al electrode was fabricated by photolithography patterning to form MOS capacitors after back Al electrode was prepared by magnetron sputtering. Atomic force microscopy (AFM, Bruker Dimension Edge, Bruker Nano Inc., Billerica, WA, USA), X-ray photoelectron spectroscopy (XPS, Thermo Scientific K-Alpha, Thermo Fisher Scientific Inc., Waltham, MA, USA) were used to character the properties of La_2_O_3_/Al_2_O_3_ nanolaminates on MoS_2_. The standard electrical measurements were performed at room temperature using the Keithley 4200SCS characterization system (Tektronix Inc., Kent, WA, USA).

## 3. Results and Discussion

**Figure 2:**
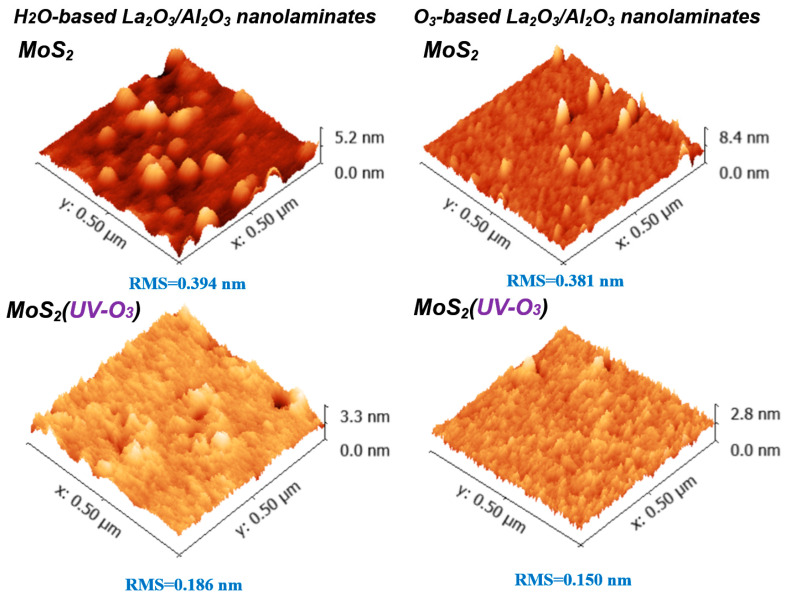
AFM results of sub-5 nm La_2_O_3_/Al_2_O_3_ nanolaminates on MoS_2_ with or without UV-O_3_ treatment.

Figure 2 shows the AFM results of La_2_O_3_/Al_2_O_3_ nanolaminates on MoS_2_. It can be found that non-uniformity surface is observed for both O_3_-based and H_2_O-based La_2_O_3_/Al_2_O_3_ nanolaminates on MoS_2_, which indicates that it is difficult to grow uniform ultrathin dielectric directly on MoS_2_. Meanwhile, a smoother surface is obtained for O_3_-based La_2_O_3_/Al_2_O_3_ nanolaminates compared to H_2_O-based La_2_O_3_/Al_2_O_3_ nanolaminates on MoS_2_. This may be explained by that, O_3_ has higher reactivity due to its strong oxidizing ability, it is easy to decompose to O_2_ and monatomic O during the ALD reactions, the monatomic O radical diffusion and desorption will significantly affect the growth of the film. Using ozone as oxidant enhances the Al_2_O_3_ film coverage and uniformity on MoS_2_ due to ozone facilitates initial TMA precursor nucleation on the MoS_2_ [20], which is consistent with the AFM results. After MoS_2_ treated with UV-O_3_, the improvement of the uniform surface is observed for both O_3_-based and H_2_O-based La_2_O_3_/Al_2_O_3_ nanolaminates (and especially for O_3_-based La_2_O_3_/Al_2_O_3_ nanolaminates). The root mean square (RMS) value of O_3_-based La_2_O_3_/Al_2_O_3_ nanolaminates decreases from 0.381 nm to 0.150 nm, while the RMS value of H_2_O-based La_2_O_3_/Al_2_O_3_ nanolaminates decreases from 0.394 nm to 0.186 nm after MoS_2_ suffered from UV-O_3_ treatment. Generally, the lack of reaction surface for MoS_2_ lead to an increase of surface roughness after ALD deposition due to the buildup of precursors and reaction products randomly occurred [11]. The improvement of uniform and decrease of RMS value suggest that the initial surface nucleation of ultrathin La_2_O_3_/Al_2_O_3_ nanolaminates on MoS_2_ can be improved by UV-O_3_ treatment.

**Figure 3:**
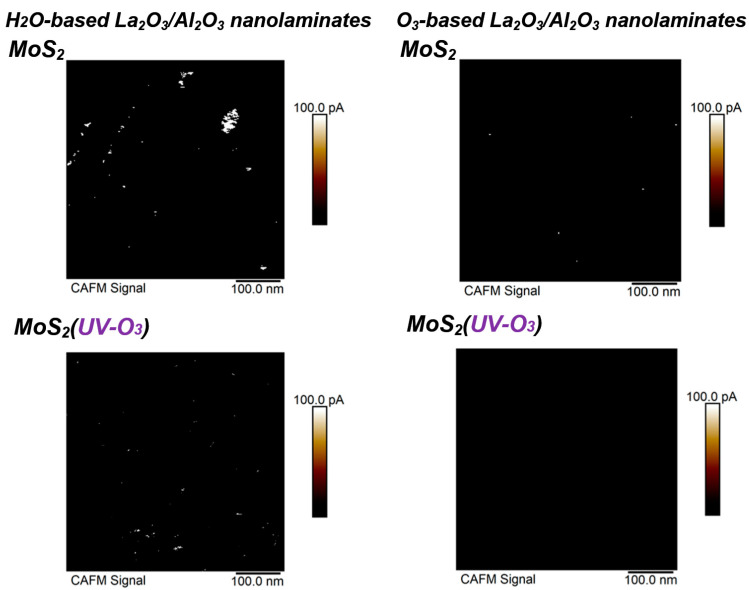
Conductive AFM images of sub-5 nm La_2_O_3_/Al_2_O_3_ nanolaminates on MoS_2_ with or without UV-O_3_ treatment.

To evaluate the dielectric electrical properties with nanometer resolution, conductive AFM measurements are carried out by applying a constant voltage between the Pt-Ir coated tip and sample. Figure 3 shows the current images measured by conductive AFM when applying a sample bias of 1 V. As shown in Figure 3, the density of leakage dots in the H_2_O-based La_2_O_3_/Al_2_O_3_ nanolaminates is higher than that in the O_3_-based La_2_O_3_/Al_2_O_3_ nanolaminates. The leakage dot is an indicator of conductive paths exist in La_2_O_3_/Al_2_O_3_ nanolaminates. They are not only attributed to surface roughness, but also possibly caused by local fluctuations in composition and/or structures, and/or by defects in La_2_O_3_/Al_2_O_3_ nanolaminates [21]. The presence of many leakage dots indicates that H_2_O-based La_2_O_3_/Al_2_O_3_ nanolaminates on MoS_2_ is not suitable for use as a gate dielectric layer. After MoS_2_ treated with UV-O_3_ treatment, the leakage dots for both O_3_-based and H_2_O-based La_2_O_3_/Al_2_O_3_ nanolaminates decreased. In particular, no leakage dot is observed for O_3_-based La_2_O_3_/Al_2_O_3_ nanolaminates. It indicates that, with the help of UV-O_3_ treatment, ultrathin O_3_-based La_2_O_3_/Al_2_O_3_ nanolaminates on MoS_2_ can serve as the gate dielectric due to its good leakage suppression properties. 

**Figure 4:**
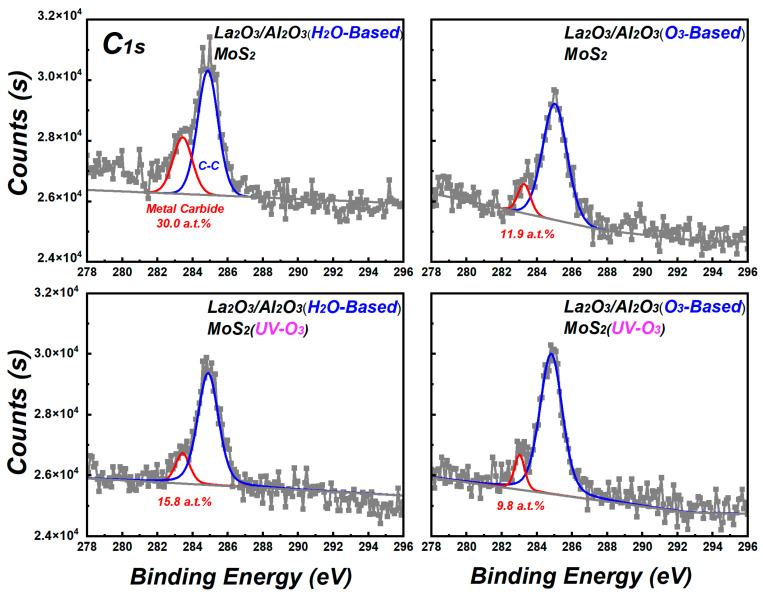
C_1s_ spectra of La_2_O_3_/Al_2_O_3_ nanolaminates on MoS_2_.

The changes in uniformity of La_2_O_3_/Al_2_O_3_ nanolaminates on MoS_2_ may be originated from the interface due to the ALD process of growing La_2_O_3_/Al_2_O_3_ nanolaminates on silicon is well established [17]. To reveal the changes that occurred at the interface, XPS measurements are performed. Figure 4 shows the C_1s_ spectra of La_2_O_3_/Al_2_O_3_ nanolaminates on MoS_2_. There are mainly two peaks in the C_1s_ spectra for all La_2_O_3_/Al_2_O_3_ nanolaminates on MoS_2_, which are located at binding energies of 283.0 eV and 284.8 eV. These peaks correspond to the metal carbide and adsorbed carbon, respectively [22]. Moreover, the peak intensity of metal carbide in H_2_O-based ALD process decreases from 30.0 a.t.% to 15.8 a.t.% after MoS_2_ suffered from UV-O_3_ treatment, while that of O_3_-based ALD process decreases from 11.9 a.t.% to 9.8 a.t.%. The lowest metal carbide content in O_3_-based La_2_O_3_/Al_2_O_3_ nanolaminates on MoS_2_ with UV-O_3_ treatment suggests that the initial interfacial reactions are greatly improved. The appearance of metal carbide is an indication that poor interface reactions occur during the ALD process, which can originate from the generation of intermediates or by-products of metal precursors. MoS_2_ suffered from low-power UV-O_3_ treatment form the weak chemical bond of S-O on the surface without hampering its electrical performance [15], which can supply the reaction interface groups at the MoS_2_ surface during the ALD deposition. As a result, the residuals of the metal carbide or its intermediate precursor during the first ALD reaction cycles can be reduced and the roughness of the nanolaminates can be improved.

**Figure 5:**
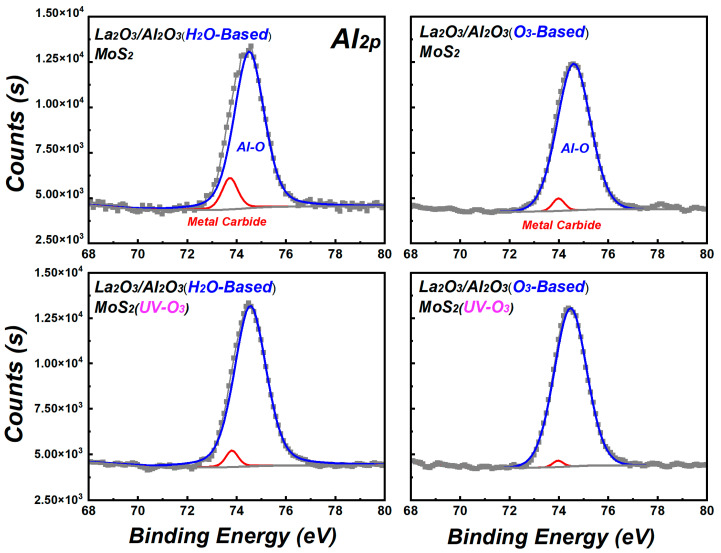
Al_2p_ spectra of La_2_O_3_/Al_2_O_3_ nanolaminates on MoS_2_.

In order to further confirm the residue in La_2_O_3_/Al_2_O_3_ nanolaminates on MoS_2_, Figure 5 shows the Al_2p_ spectra of La_2_O_3_/Al_2_O_3_ nanolaminates on MoS_2_. As shown in Figure 5, the Al_2p_ spectra can be fitted to two peaks, which located at the binding energy of ~74.6 eV and 73.8 eV, respectively. 74.6 eV belongs to the Al-O bond, and the lower 73.8 eV is related to carbide [22]. The content of carbide in H_2_O-based La_2_O_3_/Al_2_O_3_ nanolaminates decreases from 9.66 a.t.% to 3.88 a.t.% after MoS_2_ treated with UV-O_3_ treatment, while that of O_3_-based La_2_O_3_/Al_2_O_3_ nanolaminates decreases from 2.98 a.t.% to 0.92 a.t.% after MoS_2_ treated with UV-O_3_ treatment. The variation of carbide content in La_2_O_3_/Al_2_O_3_ nanolaminates on MoS_2_ is consistent with the C_1s_ results. Due to lack of dangling bonds or nucleation sites on MoS_2_, the initial reaction of ALD is dependent on weakly physical adsorbed TMA precursors on MoS_2_ surface. UV-O_3_ treatment forms the weak S-O bonds on MoS_2_ and facilitates the uniform physical adsorption of precursor, which is beneficial for the improvement of initial ALD self-limiting surface reactions. O_3_ has a stronger ability than water to split the C-H or Al-C bonds which attached to metal atoms in the deposition [18]. As a result, the concentration of metal carbide in O_3_-based La_2_O_3_/Al_2_O_3_ nanolaminates is lower than H_2_O-based La_2_O_3_/Al_2_O_3_ nanolaminates. 

**Figure 6:**
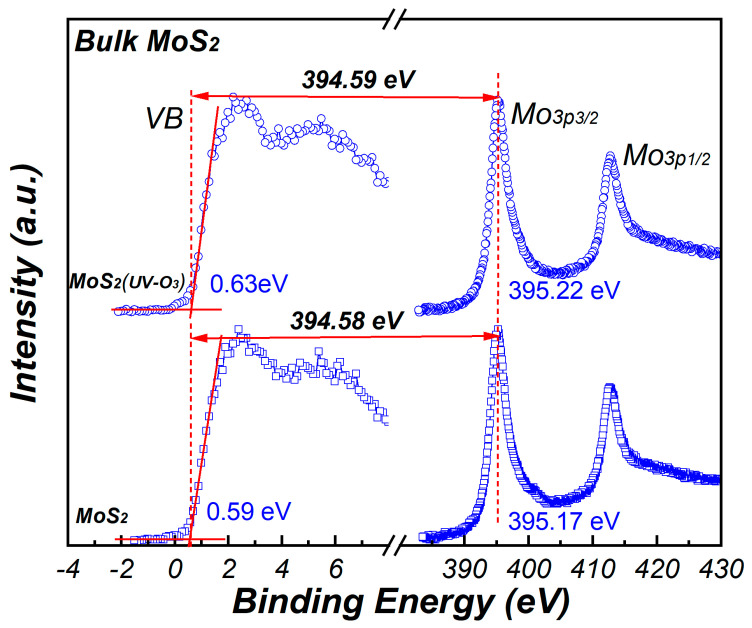
The XPS core level spectra of Mo_3p_ for MoS_2_.

To determine the valence band offset (VBO) between La_2_O_3_/Al_2_O_3_ nanolaminates and MoS_2_, the Kraut method is used which discussed in ref. [23],

(1) $$\Delta E_{VBO}={\left(E_{CL}^{Mo_{3p}}-E_{V}\right)}_{bulk,MoS_{2}}-{\left(E_{CL}^{Al_{2p}}-E_{V}\right)}_{thick\;nanolaminates}\;-{\left(E_{CL}^{Mo_{3p}}-E_{CL}^{Al_{2p}}\right)}_{thin\;nanolaminates/MoS_{2}}$$

where $E_{CL}^{Mo_{3p}}$ and$\;E_{CL}^{Al_{2p}}$ is the binding energy of the Mo_3p_ and Al_2p_ shallow core levels, respectively. *E_v_* is the binding energy corresponding to the valence band maximum (VBM). The value of VBM is determined by the intercept of the slope at the leading edge of the valence band spectrum with the base line. To correct the differential charging, the binding energy calibration was performed using a gold standard sample. Figure 6 shows the core level spectra of ~10 nm sputtered MoS_2_ with or without UV-O_3_ treatment. The energy difference between the Mo_3p_ core level and the VBM is 394.58 eV and 394.59 eV for the clean MoS_2_ and MoS_2_ treated with UV-O_3_ treatment, respectively. These values are agreed well with the values reported in ref. [24]. 

**Figure 7:**
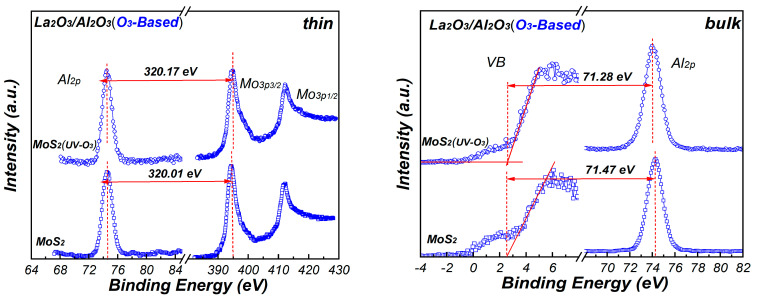

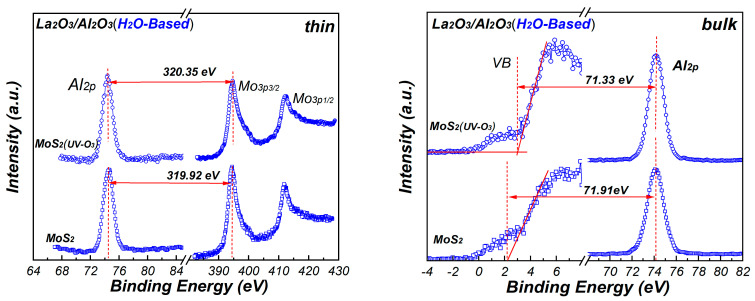
The XPS core-level and valence band spectra of thin and bulk La_2_O_3_/Al_2_O_3_ nanolaminates.

Figure 7 shows the XPS core level spectra of Mo_3p_ and Al_2p_ for La_2_O_3_/Al_2_O_3_ nanolaminates. The core level energies are obtained by curve fitting to ensure high accuracy binding energy of the peak. In order to measure the band offset between La_2_O_3_/Al_2_O_3_ nanolaminates and MoS_2_, ~10 nm H_2_O-based and O_3_-based La_2_O_3_/Al_2_O_3_ nanolaminates are prepared for use as bulk films, respectively. As shown in Figure 7, the energy difference values between the core level energies are determined. Using these energy difference values with Equation (1), the VBO values of the H_2_O-based and O_3_-based La_2_O_3_/Al_2_O_3_ nanolaminates on MoS_2_ can be derived. The VBO of 3.10 eV and 3.14 eV is obtained for O_3_-based La_2_O_3_/Al_2_O_3_ nanolaminates on MoS_2_ and MoS_2_ with UV-O_3_ treatment, respectively. In addition, the VBO of H_2_O-based nanolaminates on MoS_2_ and MoS_2_ with UV-O_3_ treatment is 2.75 eV and 2.91 eV, respectively. The results indicate that the VBO is affected by the different oxidants and UV-O_3_ treatment. The negligible VBO variations for O_3_-based La_2_O_3_/Al_2_O_3_ nanolaminates suggest that it has a better stability compared to H_2_O-based La_2_O_3_/Al_2_O_3_ nanolaminates. 

**Figure 8:**
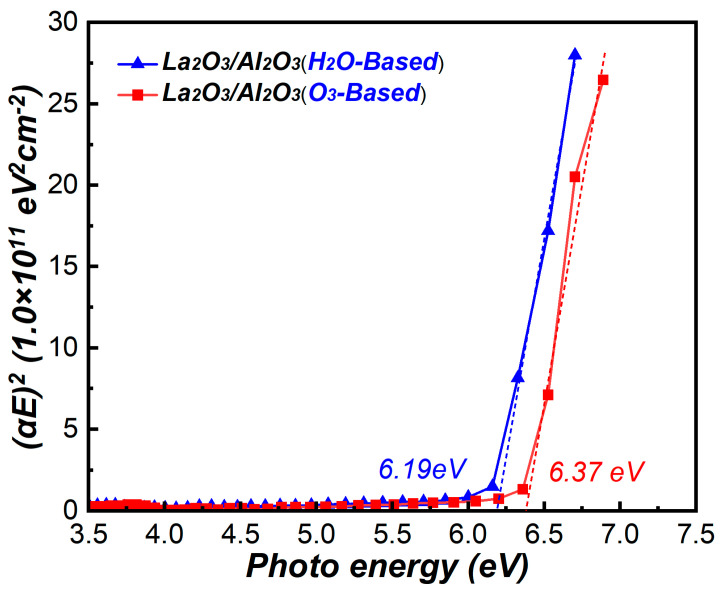
(*αE*)^2^ versus photo energy *E* of La_2_O_3_/Al_2_O_3_ nanolaminates.

To obtain the conduction band offset (CBO) between La_2_O_3_/Al_2_O_3_ nanolaminates and MoS_2_, the optical band gaps of La_2_O_3_/Al_2_O_3_ nanolaminates are measured. The optical band gaps form the plots of (*αE*)^2^ versus photo energy *E* are shown in Figure 8. The extrapolation of the linear part of (*αE*)^2^ –E down to (*αE*)^2^ = 0 gives the values of band gaps [25]. The measured band gap value of O_3_-based and H_2_O-based La_2_O_3_/Al_2_O_3_ nanolaminates is 6.37 eV and 6.19 eV, respectively. These values are in good agreement with the reported values of La_2_O_3_/Al_2_O_3_ gate stack or LaAlO_3_ films ranging from 6.1–6.4 eV [17,26]. The results indicate that the O_3_-based La_2_O_3_/Al_2_O_3_ nanolaminates has a lager bandgap value compared to the H_2_O-based nanolaminates. This may be caused by the lower content of impurities found in O_3_-based La_2_O_3_/Al_2_O_3_ nanolaminates compared to H_2_O-based nanolaminates. 

**Figure 9:**
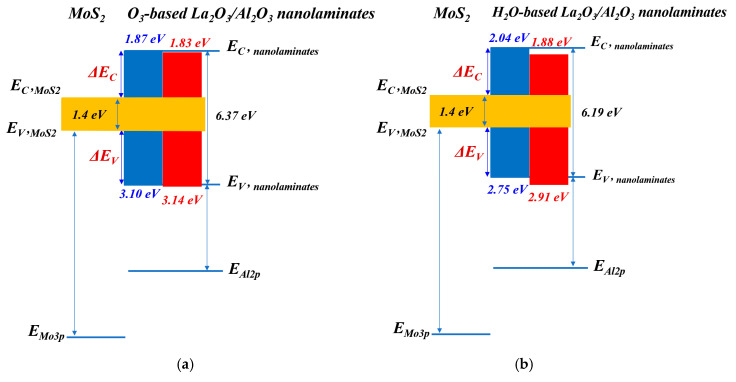
Band diagrams of (**a**) O_3_-based La_2_O_3_/Al_2_O_3_ nanolaminates and (**b**) H_2_O-based La_2_O_3_/Al_2_O_3_ nanolaminates on MoS_2_ (Blue for MoS_2_ and red for MoS_2_ treated with UV-O_3_ treatment).

Using the calculated VBO and band gap values, the conduction band offset between La_2_O_3_/Al_2_O_3_ nanolaminates and MoS_2_ can be attained by the following equation:

(2) $$\Delta E_{CBO}=E_{g}^{{\mathrm{La}}_{2}O_{3}/{\mathrm{Al}}_{2}O_{3}\;\mathrm{nanolaminates}}-E_{g}^{MoS_{2}}-\Delta E_{VBO}$$

where $E_{g}^{{\mathrm{La}}_{2}O_{3}/{\mathrm{Al}}_{2}O_{3}\;\mathrm{nanolaminates}}$ and $E_{g}^{MoS_{2}}$ is the bandgap of La_2_O_3_/Al_2_O_3_ nanolaminates and MoS_2_, respectively. The bandgap of 1.4 eV for MoS_2_ is used here [27]. According to the Equation (2), the CBO of O_3_-based La_2_O_3_/Al_2_O_3_ nanolaminates on MoS_2_ and MoS_2_ with UV-O_3_ treatment is 1.87 eV and 1.83 eV, respectively. Meanwhile, the CBO of H_2_O-based La_2_O_3_/Al_2_O_3_ nanolaminates on MoS_2_ and MoS_2_ with UV-O_3_ treatment is 2.04 eV and 1.88 eV, respectively. The corresponding band diagrams are illustrated in Figure 9. It can be seen that both La_2_O_3_/Al_2_O_3_ nanolaminates/MoS_2_ interface have a Type I alignment, where the conduction band edge and valence band edge of MoS_2_ are located within the bandgap of La_2_O_3_/Al_2_O_3_ nanolaminates. Furthermore, both CBO and VBO values of La_2_O_3_/Al_2_O_3_ nanolaminates on MoS_2_ provide excellent electron and hole barriers due to their values larger than 1 eV, ensuring La_2_O_3_/Al_2_O_3_ nanolaminates suitability for FETs applications. Remarkably, O_3_-based La_2_O_3_/Al_2_O_3_ nanolaminates has a higher VBO compare with H_2_O-based La_2_O_3_/Al_2_O_3_ nanolaminates, which is better for *p*-channel FETs application.

**Figure 10:**
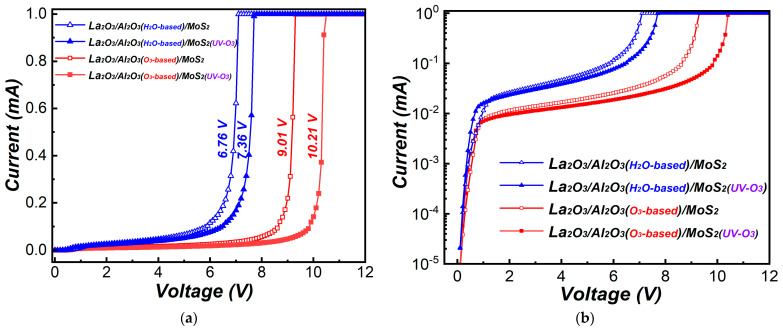
(**a**) Linear-scale and (**b**) log-scale I-V curves of MOS capacitors for La_2_O_3_/Al_2_O_3_ nanolaminates on MoS_2_.

Figure 10 shows the I-V curves of La_2_O_3_/Al_2_O_3_ nanolaminates on MoS_2_ after fabricated metal-oxide-semiconductor (MOS) capacitor. At the applied voltage of 2 V, for O_3_-based La_2_O_3_/Al_2_O_3_ nanolaminates, the leakage current decreased from 1.2 × 10^−2^ mA to 9.6 × 10^−3^ mA, while the breakdown voltage increased from 9.01 V to 10.21 V after MoS_2_ treated with UV-O_3_ treatment. The same trend is observed in H_2_O-based La_2_O_3_/Al_2_O_3_ nanolaminates. The leakage current decreased from 2.6 × 10^−2^ mA to 2.3 × 10^−2^ mA, while the breakdown voltage increased from 6.76 V to 7.36 V after MoS_2_ treated with UV-O_3_ treatment. The breakdown voltage is obtained when the leakage current reaches 1 mA [28]. The decease of leakage current and increase of breakdown voltage may be attributed to the uniformity of the La_2_O_3_/Al_2_O_3_ nanolaminates as well as the reduction of impurities or residuals at the interface. The leakage current may originate either Poole-Frenckel or Fowler-Nordheim mechanism from the point of view of quantum tunneling [29,30], which has been confirmed in our measurement. The lowest leakage current and highest breakdown voltage are obtained for O_3_-based La_2_O_3_/Al_2_O_3_ nanolaminates on MoS_2_ with UV-O_3_ treatment, making it a promising dielectric candidate for the application of MoS_2_ FETs.

## 4. Conclusions

In this study, atomic layer deposition growth of sub-5 nm La_2_O_3_/Al_2_O_3_ nanolaminates on MoS_2_ using different oxidants (H_2_O and O_3_) and the UV-O_3_ pretreatment on MoS_2_ are investigated. Compared with H_2_O-based La_2_O_3_/Al_2_O_3_ nanolaminates on MoS_2_, better uniformity and lower leakage dots were observed for O_3_-based La_2_O_3_/Al_2_O_3_ nanolaminates on MoS_2_. This is associated with the metal carbide concentration in La_2_O_3_/Al_2_O_3_ nanolaminates on MoS_2_, which is generated by insufficient interfacial reactions. UV-O_3_ treatment can decrease the residuals of the metal carbide and improve the deposition of La_2_O_3_/Al_2_O_3_ nanolaminates on the MoS_2_ interface by introducing the weak S-O bonds to MoS_2_ surface, leading to the properties of La_2_O_3_/Al_2_O_3_ nanolaminates being substantially improved. The band offset values of both O_3_-based and H_2_O-based La_2_O_3_/Al_2_O_3_ nanolaminates/MoS_2_ are larger than 1 eV, which can provide eligible electron and hole barrier height. In particular, a higher valence band offset is obtained for O_3_-based La_2_O_3_/Al_2_O_3_ nanolaminates compared to H_2_O-based La_2_O_3_/Al_2_O_3_ nanolaminates. Consequently, O_3_-based La_2_O_3_/Al_2_O_3_ nanolaminates on MoS_2_ exhibits smaller leakage current and higher breakdown voltage, especially after MoS_2_ suffered from UV-O_3_ treatment. All results indicate that O_3_-based La_2_O_3_/Al_2_O_3_ nanolaminates on MoS_2_ with UV-O_3_ treatment is a more appropriate process to obtain sub-5 nm uniform La_2_O_3_/Al_2_O_3_ nanolaminates on MoS_2_ due to its good electrical characteristics, providing important implications for its integration into transistors.

## Data Availability

Data will be made available upon reasonable request.
